# Hospitalizations for varicella in children and adolescents in a referral hospital in Hong Kong, 2004 to 2008: A time series study

**DOI:** 10.1186/1471-2458-11-366

**Published:** 2011-05-23

**Authors:** Johnny YC Chan, Linwei Tian, YW Kwan, WM Chan, CW Leung

**Affiliations:** 1 Department of Pediatrics and Adolescent Medicine, Princess Margaret Hospital, Hong Kong SAR, China; 2 School of Public Health and Primary Care, the Chinese University of Hong Kong, Hong Kong SAR, China

**Keywords:** Varicella, Chickenpox, Time series study, Child, humidity, cool season

## Abstract

**Background:**

Varicella accounts for significant morbidities and remains a public health issue worldwide. Climatic factors have been shown to associate with the incidence and transmission of various infectious diseases. We describe the epidemiology of varicella in paediatric patients hospitalized at a tertiary referral hospital in Hong Kong from 2004 to 2008, and to explore the possible association between the occurrence of varicella infection and various climatic factors.

**Methods:**

The hospital discharge database of Princess Margaret Hospital was retrospectively analyzed for admissions associated with varicella from 2004 to 2008. Meteorological data were obtained from the monthly meteorological reports from the Hong Kong Observatory website. Time series analysis was performed with Poisson regression using a Generalized Estimating Equation (GEE) approach.

**Results:**

During the study period, 598 children were hospitalized for varicella. The mean age on admission was 57.6 months, and the mean duration of hospitalization was 3.7 days. The overall complication rate was 47%. The mean monthly relative humidity, especially in cool seasons, was inversely correlated with the monthly varicella cases of the same month.

**Conclusions:**

Varicella can lead to serious complications and prolonged hospitalization, even in previously healthy children. Lower relative humidity in cool seasons is associated with higher number of paediatric varicella hospital admissions. These findings are useful for a better understanding of the pattern of paediatric varicella hospitalization in Hong Kong.

## Background

Varicella, or chickenpox, is an infectious disease caused by the ubiquitous varicella-zoster virus (VZV). It used to be considered a relatively benign communicable disease of childhood [1]. However, serious complications can occur, including secondary bacterial skin and soft tissue infections, cerebellitis, encephalitis, pneumonia and coagulopathy [1,2]. Hospitalization rates due to chickenpox are considerably high in developed countries, especially among children. The reported chickenpox complication rates range from 40.7% to 83.3% of children hospitalized with the condition in various studies [3-5]. Furthermore, a mortality rate of 2-3 per 100,000 affected persons has been reported [1]. Therefore, varicella remains an important public health issue worldwide. Despite the availability of a safe and effective varicella vaccine since 1986, there is no recommendation for universal vaccination against varicella in Hong Kong [6-8].

It has been reported that the epidemiology of varicella appears to vary among different geographic regions, climatic belts, population densities, and degrees of socioeconomic development [9-11]. A number of studies have been conducted to investigate the possible association between the occurrence of varicella infection and various environmental factors, such as temperature, rainfall and humidity. Wu et al has shown that season and temperature are significantly related to varicella incidence in Taiwan [12]. Another study performed by Kokaze et al demonstrated that the annual temperature variation affected the seasonal variations of varicella incidence in Japan [13]. In Hong Kong, the rates and characteristics of chickenpox in children have not been clearly described.

The objective of this study was to investigate the local epidemiology and seasonal trend of varicella in hospitalized children in Hong Kong. Furthermore, the possible underlying association between varicella incidence and various climatic factors was also explored using time series methodology.

## Methods

A retrospective study was conducted to analyse the clinical information of all children and adolescents aged below 18 years who were hospitalized for varicella at Princess Margaret Hospital, a tertiary referral hospital for paediatric infectious diseases, between 1 January 2004 and 31 December 2008. Cases were identified by reviewing hospital discharge records retrieved using the relevant International Classification of Diseases 9^th ^Edition (ICD-9) principal or secondary discharge diagnostic codes for varicella or chickenpox, as in other epidemiological studies [2,3]. The inclusion criteria were: children aged below eighteen years; principal or secondary discharge diagnostic codes being either varicella or chickenpox; and patients being admitted for varicella to Princess Margaret Hospital during the period from 1 January 2004 to 31 December 2008. All hospital admissions, not limited to emergency admissions, were included for time series analysis. Cases of herpes zoster were excluded.

Hospital discharge records for all the cases fulfilling the inclusion criteria were retrieved. Relevant demographic, clinical and admission data were extracted from the hospital discharge records. Relevant demographic data included age and gender of the patients. Relevant clinical data included the presence or absence of associated complications and the type of complications of the patients. Important admission data included the month and year of hospital admission and the length of hospitalization. The diagnosis of encephalitis was based on clinical features (decreased consciousness and seizure or focal neurologic deficit), cerebrospinal fluid pleocytosis, electroencephalographic changes and neuroimaging findings [14]. The diagnosis of cerebellitis was based on clinical findings of ataxia and other cerebellar signs. The diagnosis of pneumonia is based on clinical, laboratory and radiological features [14]. The diagnosis of skin and soft tissue infections was based on clinical signs (cellulitis, skin abscess, myositis or necrotizing fasciitis), and surgical scarlet fever was based on clinical features (sandpaper-like erythema, strawberry tongue and Pastia's lines).

Meteorological data including monthly mean air temperature, monthly mean relative humidity and monthly total rainfall were obtained from the overall monthly reports of meteorological observations during the study period from the website of Hong Kong Observatory [15].

### Statistical analysis

Age of the patients (months) and length of hospitalization (days) were expressed as means, medians and ranges. Comparison among groups was performed using the chi-square or Fisher's exact test, where appropriate. Mann-Whitney test was used for non-normally distributed data.

Spearman rank correlation tests were performed to examine the relationship between monthly varicella incidence and climatic variables, including monthly mean temperature, monthly mean relative humidity and monthly rainfall, with a lag of zero to two months. The weather variables found to be correlated with varicella incidence were included in univariate and multivariate regression models. The monthly varicella incidence was modeled using a generalized estimating equations (GEE) approach, with a Poisson distribution. This model enables both specification of an over-dispersion term and a first-order autoregressive structure that accounts for the autocorrelation of monthly numbers of varicella cases. Besides the meteorological factors, the autoregression term ylag (number of varicella cases at one month lag) was also included in the model. As GEE are not a full likelihood-modeling method, the quasi-likelihood based information criterion (QICu) developed by Pan was computed to select the most parsimonious model [16]. When using QICu to compare two models, the model with the smaller statistic was preferred. Model with fewest parameters was preferred.

A p-value < 0.05 will be considered statistically significant. Data were analysed using SPSS software (version 16.0; SPSS Inc, Chicago, IL, USA) and SAS software (version 9 for Windows; SAS Institute, Inc., Cary, North Carolina).

## Results

### Hospitalization figures for current study

**Figure 1:**
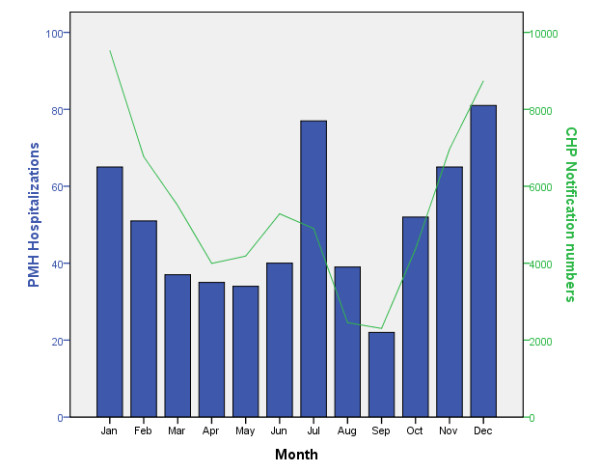
Monthly distribution of varicella-associated paediatric hospitalizations at Princess Margaret Hospital and monthly notification of varicella for all ages at Centre for Health Protection (CHP) in Hong Kong from 2004 to 2008.

In total, 604 hospital records with discharge diagnostic coding for either varicella or chickenpox were reviewed. Six cases were excluded according to the exclusion criteria, and 598 cases were included in this study for analysis. The annual varicella-associated hospitalization figures from 2004 to 2008 ranged from 83 to 156 per year. The monthly distribution of varicella-associated paediatric hospitalizations at Princess Margaret Hospital and the monthly varicella notification for all ages at Centre for Health Protection (CHP) in Hong Kong are shown in Figure 1[17]. A bimodal distribution of cases was observed, with hospitalizations peaking in the winter months of December and January, as well as in the summer months of June and July.

### Demographic features

There were a total of 328 male (54.8%) and 270 female (45.2%) patients hospitalized for varicella, with a slight male predominance at a ratio (1.2 : 1). The mean age was 57.6 months (range 1-204 months). 64 patients (10.7%) were aged 6 months or under, 335 (56.0%) were aged 7 months to 5 years, 126 (21.1%) were aged above 5 years to 10 years, and 73 (12.2%) were aged above 10 years.

### Length of hospitalizatio

The overall mean length of hospitalization was 3.7 days (range 1-27 days). It was noted that the length of hospitalization was significantly longer in patients with complicated varicella compared to those without complications, with a mean of 4.3 days (range 1-24 days) vs 3.1 days (range 1-27 days), respectively (p < 0.001).

### Complications of varicella

Among all cases, 281 out of 598 (47%) patients developed complications of varicella. It was found that skin and soft tissue infections were the most commonly occurred complication, accounting 121 out of 281 patients (43.1%) who developed complicated varicella. Ninety-nine patients (35.2%) had surgical scarlet fever. Neurological complications were observed in 51 patients (18.1%), including one case of encephalitis, one case of aseptic meningitis, four cases of cerebellitis, five cases of afebrile seizure and 40 cases of febrile seizure. Pneumonia was identified in 23 patients (8.2%). Nine patients (3.2%) developed other complications, including clinical sepsis, immune thrombocytopenic purpura and Henoch-Schonlein purpura in 4, 3 and 2 patients, respectively. There were five patients with severe complicated varicella requiring intensive care but there was no fatal case. The mean age of patients with complicated varicella was 51.6 months (range 1-204 months), which was not significantly different from those without complications (mean age of 62.9 months, range 1-204 months, p = 0.183).

### Periodicity and climatic factors variation

**Figure 2:**
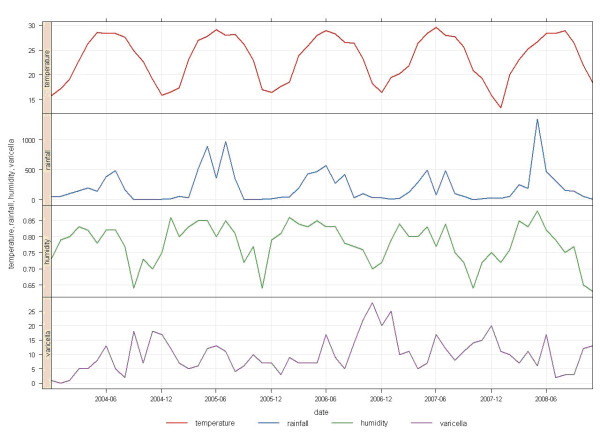
The monthly mean air temperature, total rainfall, mean relative humidity, and the monthly distribution of chickenpox cases in Princess Margaret Hospital from 2004 to 2008*. (* The units for the monthly mean temperature and monthly rainfall are degrees Celsius and millimeters respectively. For the relative humidity, 0.75 refers to 75% relative humidity, and 0.80 refers to 80% relative humidity, etc. For the dates, 2004-06 denotes June 2004, and 2004-12 denotes December 2004, and so on).

The monthly distribution of chickenpox cases in Princess Margaret Hospital from 2004 to 2008 is shown in Figure 2. The periodicity of various climatic factors, namely monthly mean air temperature, monthly mean relative humidity and monthly rainfall, are also shown. The units for the monthly mean temperature and monthly rainfall are degrees Celsius and millimeters respectively. For the relative humidity, 0.75 refers to 75% relative humidity, and 0.80 refers to 80% relative humidity, etc. For the dates, 2004-06 denotes June 2004, and 2004-12 denotes December 2004, and so on.

Table 1 shows the Spearman correlation analysis for the relationship between numbers of varicella cases (2004 - 2008) and climatic variables with a lag of zero to two months. Monthly mean relative humidity was inversely correlated with varicella cases at lags of zero and one month; monthly total rainfall was inversely correlated with varicella cases of the same month.

**Table 1:** Coefficients of Spearman correlation analysis between varicella cases (2004-2008) at Princess Margaret Hospital and climatic variables (* P < 0.05)

Lag months	Tmean	Humid	Rain
0	-0.122	-0.262*	-0.294*
1	-0.014	-0.280*	-0.176
2	0.069	-0.253	-0.097

Tmean = Monthly mean air temperature

Humid = Monthly mean relative humidity

Rain = Monthly total rainfall

Table 2 shows the best fitting model, with the smallest QICu value (QICu = -589.5), to characterize the relationship between monthly varicella cases and weather variables. The number of varicella cases was first-order autoregressive as the number of varicella cases in the current month was related to that in the previous month (Beta = 0.038, 95% confidence interval 0.019, 0.057, P < 0.0001). Monthly mean relative humidity had a negative effect on the number of varicella cases of the same month (Beta = -2.67, 95% confidence interval -4.34, -1.01, p = 0.002). Forcing average temperature and total rainfall into the model did not change the coefficient estimates for relative humidity significantly.

**Table 2:** Result for best fitting model with GEE analysis

	Beta	Standard error	p-value
ylag	0.038	0.0096	<0.0001
Humid0	-2.67	0.848	0.002
QICu		-589.5	

ylag = Number of varicella cases at lag of one month

Humid0 = Mean relative humidity at the same month

To check whether the residual was a white noise or not, Q test was performed with no significant time trend identified in the residual of the proposed model (Q statistics to a lag of 6 = 3.35, p = 0.76). In addition, Shapiro-Wilk test was performed and confirmed the normality of the residual (Shapiro-Wilk statistics = 0.97, p = 0.10). The model that adjusted for first-order autocorrelation was written as:

Where Y, ylag and Humid0 stand for number of varicella cases in that month, number of varicella cases in the previous one month (at lag of one) and monthly mean relative humidity in the same month. The monthly mean relative humidity of the same month was found to be a predictor for the monthly varicella cases.

We also conducted a stratified analysis by two seasons (October-March defined as cool seasons and the other months hot season) to see how the effect of humidity varies. Humidity remained a significant predictor of varicella in the cool season model (Beta = -3.02, 95% confidence interval -5.47, -0.58, p = 0.0152), while it was not significant in the hot season model.

## Discussions

This is the first time series study exploring the possible assoication between paediatric varicella hospitalization and weather factors in Hong Kong. Our dataset is relevant because the involved hospital, Princess Margaret Hospital, is a tertiary referral hospital for paediatric infectious diseases. The involved hospital received not only patients in its locality, but also patients transferred from other public hospitals. Age-specific hospitalization rates vary between studies and generally peak in children aged 1-4 years as they did in the present study [3,4,18]. The complication rates reported in literature among children hospitalized for varicella infection range from 40.7% to 83.3% [3-5]. In the present study, the complication rate was 47%. The distribution of different complications in this study was similar to that reported in other paediatric series, with skin and neurological complications occurring most commonly [4,5,14,18].

Time series analysis has been used extensively to study the effect of weather factors on various infectious diseases [19-23]. In the current study, Poisson regression with generalized estimating equations (GEE) was used to examine the potential effect of various weather factors on the incidence of chickenpox in paediatric patients hospitalized in a tertiary hospital in Hong Kong during the period of 2004 to 2008. Monthly mean relative humidity, especially in cool seasons, was found to be inversely correlated with the number of varicella cases in the same month.

It has been suggested that the transmission potential of the VZV virus might be adversely affected by a combination of high ambient temperatures and humidity in tropical regions [24]. A review of data from Southeast Asia showed that the peak incidence of varicella infection occurred during cooler months and in cooler, more temperate regions [25]. Outbreaks of varicella appear to be more common in the cooler months of the year in India and in Thailand; incidence rates are highest in the temperate northern region of the country [26-28]. Data from studies outside Southeast Asia also support the notion of reduced VZV transmission in hot, humid climates [29]. For example, Maretic and Cooray found that the number of chickenpox admissions to a regional hospital in the cool, dry season more than doubled compared to the hot monsoon period in their study of a Sri Lankan population [30]. Lolekha et al reported that the age-adjusted seroprevalence was significantly higher in the cooler than in the warmer regions in Thailand [31]. Wu et al has shown that season and temperature are significantly related to varicella incidence in Taiwan [12]. Another study performed by Kokaze et al demonstrated that the annual temperature variation affected the seasonal variations of varicella incidence in Japan [13].

Varicella patients excreted the virus from their respiratory tract or vesicles and disseminated the virus to the environment via an aerosol route [32]. VZV can be transmitted via airborne spread [33]. However, the exact mechanism for the potential association between relative humidity and varicella incidence and transmission is not known. One could hypothesize that with a lower relative humidity, the density of air particles would be lower and hence the VZV could have stayed longer in the air during its airborne transmission to a long distance. Another possibility could be that in a dry environment with more dry skin, the patients with chickenpox could suffer from excessive itchiness where more skin scratching would facilitate more viral spread to the environment. In addition, the more common respiratory symptoms such as cough and sputum production in the dry season could enhance the aerosol spread of the VZV. However, all these hypotheses warrant further studies to explore the mechanism underlying the association between relative humidity and varicella infection.

Our study may have some limitations. First, it is based on hospitalization discharge records, and therefore children with mild disease and complications may have been excluded due to the absence of hospitalization. The potential climatic association with varicella might have been dampened by this selective sample. Second, using discharge diagnoses codes for data collection might result in errors due to the possibility of miscoding. But studies investigating the accuracy of ICD-9 codes for various diseases in different countries suggested that this is a useful tool for epidemiological studies [4,34,35]. In addition, chickenpox is considered to be a easily recognisable disease [2,36]. Third, as the weather fluctuation in Hong Kong is relatively small, the potential effect of weather factors might have been better captured by decreasing the data interval, for instance, a weekly or daily basis, or increasing the study period. It should be acknowledged that varicella transmission is multifactorial [37-39]. Apart from potential weather attributes, other environmental and host factors may also affect the incidence and transmission of the disease. Further studies to incorporate more variables such as age, geographic location, and frequency of contact expressed by population density and school contact patterns are warranted in the future.

## Conclusions

In conclusion, varicella is a disease that can provoke serious complications and long hospital stays, even in healthy children. Lower relative humidity, especially in the cool seasons, is associated with higher number of paediatric varicella hospital admissions. These findings are useful for a better understanding of the pattern of paediatric varicella hospitalization in Hong Kong.

## Conflict of interest statement

The authors declare that they have no competing interests.

## Authors' contributions

JYCC conceived of the study and drafted the manuscript. LT participated in the design of the study and performed the statistical analysis. YWK and WMC participated in the study design and data collection. CWL initiated and coordinated the study. All authors read and approved the final manuscript.

## Pre-publication history

The pre-publication history for this paper can be accessed here:

http://www.biomedcentral.com/1471-2458/11/366/prepub
